# Testing models at the neural level reveals how the brain computes subjective value

**DOI:** 10.1073/pnas.2106237118

**Published:** 2021-10-22

**Authors:** Tony B. Williams, Christopher J. Burke, Stephan Nebe, Kerstin Preuschoff, Ernst Fehr, Philippe N. Tobler

**Affiliations:** ^a^Zurich Center for Neuroeconomics, Department of Economics, University of Zurich 8006 Zurich, Switzerland;; ^b^Delta Nine Behavioral Economics, Bellevue, WA 98004;; ^c^Geneva Finance Research Institute, University of Geneva, Geneva 1211, Switzerland; and; ^d^Zurich Center for Neuroscience, University of Zurich and ETH Zurich, Zurich 8057, Switzerland

**Keywords:** risk taking, physiological foundation of behavior, neural valuation systems, neuroeconomics

## Abstract

Decisions are based on the subjective values of choice options. However, subjective value is a theoretical construct and not directly observable. Strikingly, distinct theoretical models competing to explain how subjective values are assigned to choice options often make very similar behavioral predictions, which poses a major difficulty for establishing a mechanistic, biologically plausible explanation of decision-making based on behavior alone. Here, we demonstrate that model comparison at the neural level provides insights into model implementation during subjective value computation even though the distinct models parametrically identify common brain regions as computing subjective value. We show that frontal cortical regions implement a model based on the statistical distributions of available rewards, whereas intraparietal cortex and striatum compute subjective value signals according to a model based on distortions in the representations of probabilities. Thus, better mechanistic understanding of how cognitive processes are implemented arises from model comparisons at the neural level, over and above the traditional approach of comparing models at the behavioral level alone.

## Significance

In recent years, models have played an increasingly important role for understanding the brain in cognitive, behavioral, and systems neuroscience. Decision neuroscience in particular has benefitted greatly from the application of economic models of choice preferences to neural data. However, an often-overlooked aspect is that many models of preferences have a generic problem—they make extremely similar behavioral predictions. Here, we demonstrate that to understand the mechanisms of valuation in the brain, it is useful to compare models of choice preferences not only at the behavioral but also at the neural level.

Psychology, economics, and other social sciences develop competing models and theories to explain how we make choices. However, the capacity to select the one model that explains behavior best is limited by the fact that different models often make similar predictions about behavior. Therefore, it can be difficult to distinguish between models based on behavioral data alone. To illustrate, one landmark study applied 11 different models of subjective valuation to participants’ choices between different lotteries in an attempt to identify the model that best represented each participant’s preferences (1). Even though different models explained behavior better at the individual level, on average the models made the same predictions on over 90% of decisions across two experimental datasets, suggesting that the prediction similarity of competing models is pervasive. The prediction similarity is particularly striking given that the models make vastly different assumptions about underlying processes (see last paragraph of the Introduction). Moreover, these difficulties of model selection are not limited to the realm of value-based decision-making but emerge in various areas of behavioral research on, for example, learning (2), memory (3), and perception (4). Informing the model selection process by neuroscientific data is one possible solution for this problem.

Computational and decision neurosciences aim to characterize the neural mechanisms implementing cognitive functions and test if existing behavioral models accurately describe neural processes. Typically, competing models are fitted to behavioral data and their likelihoods or amounts of explained variance are compared. The winning model is then used to generate estimates of unobservable, i.e., latent, variables (e.g., subjective values or prediction errors), and activity correlating with these variables at the neural level is used to conclude that the brain implements that model (5, 6). However, the insights that can be gained with this approach are severely limited if different models predict similar behavior or correlated latent variables at the neural level.

Here, we addressed this problem by testing whether brain activity can be directly used to compare and select between competing theories with similar behavioral predictions. That is, even if competing theories make similar behavioral predictions and identify the same brain regions, are the neural computations in these regions differentially captured by the different theories? By asking this question and performing model comparison at the neural level, we deviate from the standard practice in model-based neuroscience of first fitting different models to behavioral data and then using the behaviorally best-fitting model to analyze the neural data. This procedure, if successful, would be applicable also to other areas of behavioral, cognitive, and clinical neuroscience, which use computational models to explain functions such as reinforcement learning, perceptual decision-making, and psychiatric disorders.

We performed model comparison in the context of value-based decision-making and scanned participants while they chose between lotteries with a wide range of magnitudes and probabilities. These lotteries were specifically designed to differentiate between different models of choice preference (7). We compared three major decision theories (expected utility [EU] theory, prospect theory [PT], and the mean-variance-skewness [MVS] model), which are all consistent with the idea that risky decisions are based on assigning subjective values to risky choice alternatives (8–11). EU essentially proposes that choice preferences can be represented by summing up probability-weighted subjective values (utilities) of outcomes, using objective probability (see *Experimental Procedures* for details of all three models). Like EU, PT also employs a mechanism of weighting subjective values of outcomes with probabilities. It additionally assumes that probability is processed subjectively (typically overweighting small and underweighting large probabilities), that outcomes become gains or losses in relation to a reference point, and that losses weigh more heavily than gains of equal absolute size. In contrast, the MVS model suggests that choice preferences can be represented by a linear combination of individually weighted summary statistics of outcome distributions (12–16). Thus, computations of subjective value and implied processes differ between these models. At the formal level, they are either nested or equivalent under specific conditions (17). Moreover, all of the models have been successfully used to explain behavior (18), and attempts to adjudicate between models based on behavior alone yielded conflicting results (1, 19).

## Results

**Fig. 1.**
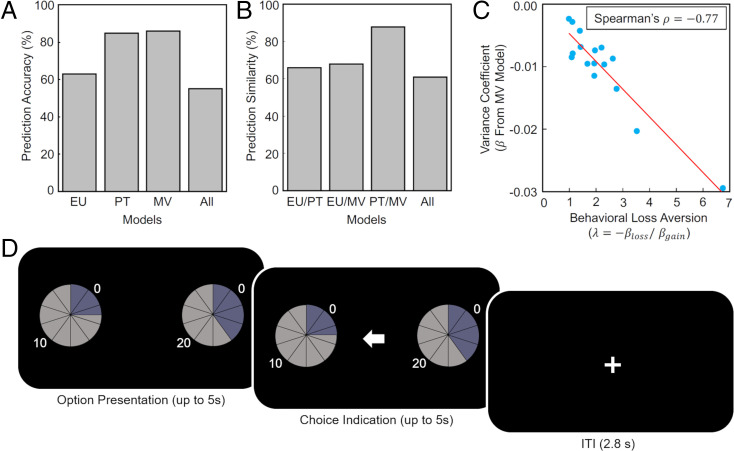
Model accuracy and similarity for data from Tom and colleagues (20) and task design. (*A*) EU theory predicted the correct choice by participants of the study by Tom and colleagues (20) on 63% of all trials, PT on 85%, and mean-variance (MV) on 86% (as probabilities were fixed at 0.5, skewness preferences could not be assessed). All three models made a correct prediction on 55% of trials. (*B*) Model similarity (i.e., instances where models make the same choice prediction on a trial, irrespective of whether the prediction is correct) showed that PT and MV models make strikingly similar behavioral predictions (88% of all trials). (*C*) A potential reason for PT/MV similarity is that variance preferences as modeled by MV are highly correlated with the loss aversion parameter from PT (λ, computed as the ratio between the regression coefficients for gain and loss outcomes from lotteries). (*D*) Example trial from our task. Participants viewed two binary lotteries with outcomes and corresponding probabilities displayed on a pie chart. Specifically, lotteries took the form *p* chance of *x1*, 1-*p* chance of *x2*, with *x* ranging from 0 to 50 points (1 point = 0.25 CHF) and *p* ranging from 0 to 1 in smallest increments of 0.05. Participants chose a lottery using the button box, and their choice was indicated. No feedback was given regarding the outcome of the lottery. During the intertrial interval (ITI), a fixation cross was displayed.

We asked whether prediction similarity matters for neuroscientists interested in the neural mechanisms of decision-making. In addition to analyzing our own data, we therefore first reanalyzed the behavioral choice data from a landmark study on risky decision-making (20), which was the target of a wider replication effort [https://www.narps.info/ (21)]. This study approached the neural implementation of subjective valuation ex ante from the perspective of PT. We fitted EU, PT, and mean-variance models (because the lottery outcomes had fixed probabilities of 0.5, we could not assess skewness preferences) to the choice data and assessed if the different models accurately predict choices on a trial-by-trial basis. We found that PT and mean-variance models had similar predictive accuracy (85 and 86%, respectively) for participant choices (Fig. 1*A*). Strikingly, PT and mean-variance models predicted the same choice on 88% of all trials (Fig. 1*B*), arising from a significant correlation between the behavioral loss aversion parameter from PT with the variance coefficient from the mean-variance model (Fig. 1*C*; Spearman’s ρ = −0.77, *P* < 0.001). Thus, any neural regions correlating with behavioral loss aversion determined by PT are also likely to be correlated with the variance coefficient determined by the mean-variance model. EU predicted choice accurately in 63% of trials. Importantly, the similarities in choice prediction of EU with both PT and mean-variance models also were substantial (66 and 68%, respectively). The similar predictive power of competing models of value-based choice further reinforces the notion that insights about how the brain computes subjective value may be facilitated by model comparison at the neural level.

**Fig. 2.**
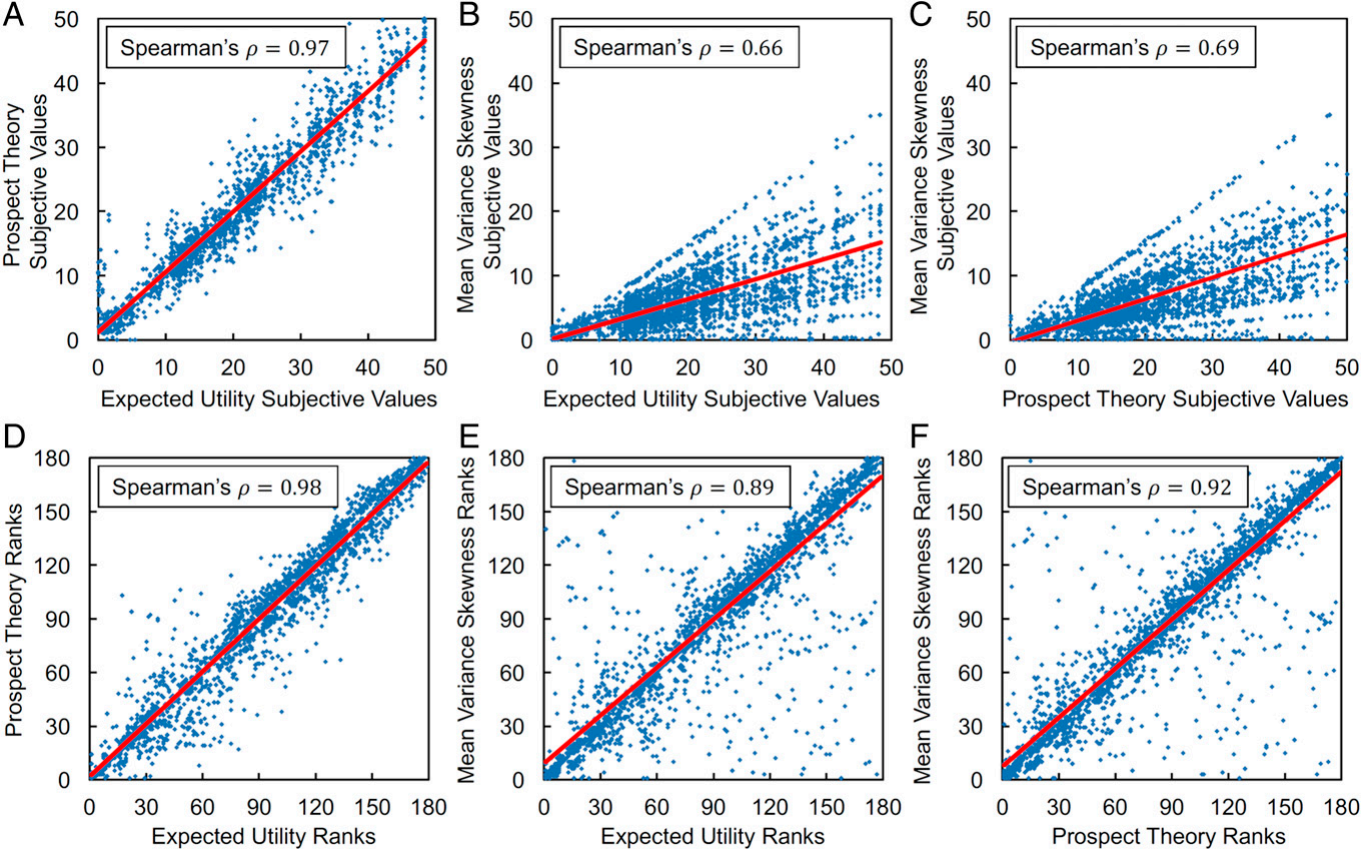
Correlations of subjective values and ordinal ranks from EU, PT, and MVS models. Using the best-fitting parameter values from the EU, PT, and MVS models, we computed the subjective values of each lottery shown to participants during the task. (*A*) Correlation between EU and PT subjective values (*B*) between EU and MVS subjective values and (*C*) between PT and MVS subjective values were all high. Moreover, ordinal rankings of lotteries were even more strongly correlated (*D*–*F*), meaning that if ordinal rankings are used to form the basis of preferences, all three models will predict very similar decisions.

Next, we tested whether the issue of prediction similarity also arose in our task (Fig. 1*D*), which used lotteries specifically designed to dissociate between models (7). Specifically, the lotteries covered a large range of probabilities, variances, and skews, allowing for probability distortion (PT) and variance-skewness parameters to make a difference to expected utility theory. We then fit the EU, PT, and MVS models to participants’ behavioral choices using maximum likelihood estimation. On the group level, the EU model correctly predicted the participants’ choices with 68% accuracy, the PT model with 74%, and the MVS model with 72%. All three models made the same choice prediction on 73% of trials. Accordingly, when we compared model predictions in a pairwise manner, the models predicted the same choices (and therefore preferences) to a high degree (EU/PT: 79%; EU/MVS: 79%; PT/MVS: 87%). Moreover, the absolute difference in the values of the two choice options negatively correlated with response times in all three models (EU: ρ = −0.171, PT: ρ = −0.198, MVS: ρ = −0.186, all *P*s < 0.001). To further assess model similarity, we used the best-fitting parameters from each participant’s EU, PT, and MVS model fits and computed the subjective values of each available lottery at the individual level. As expected, the cardinal subjective values predicted by each model were strongly correlated, with Spearman’s ρs = 0.66 to 0.97 (all *P*s < 0.001; Fig. 2 *A*–*C*). Since standard economic theory posits that the ordinal ranking of options (rather than cardinal subjective values) forms the basis of preferences (8, 22), we also calculated the ordinal ranks of all the lotteries according to each model. Strikingly, the similar behavioral predictions of the different models became even more apparent when considering the ranks of choice alternatives (Spearman’s ρs = 0.89 to 0.98; all *P*s < 0.001; Fig. 2 *D*–*F*), suggesting that all three models predict almost identical preferences if the ordinal ranks of options are considered to drive choices.

To extract neural data for model comparison, we first investigated whether some brain regions would commonly process subjective value according to all three models. How subjective value signals are encoded at the neural level during decision-making depends on the phase of the choice process (23). At least two distinct forms of subjective value signals can be distinguished. When choice options are presented, neural theories of decision-making assume that decision-makers assign a subjective value to each of them (24). Summing these subjective values up corresponds to the first form of subjective value signals (i.e., the value of the state induced by the choice options). Then, the subjective values of the choice options are assumed to be compared, resulting in differences between subjective values, which correspond to the second form of subjective value signals (25). Note that value difference is highly correlated with the difference between chosen and unchosen subjective value, which is sometimes used to characterize value-based comparison (25, 26). To capture both forms of subjective value, we assessed representations of subjective state value (summed option values) and subjective value differences, computing a general linear model (GLM) for each of the three behavioral models per participant (see *Experimental Procedures*). The GLM assessed the summed subjective values of the options from trial onset to decision time with a parametric modulator of the sum of subjective values of the two lotteries on the screen. A second parametric modulator assessed the absolute difference between the subjective values of the two lotteries with a parametric modulator at the time of the decision itself.

**Fig. 3.**
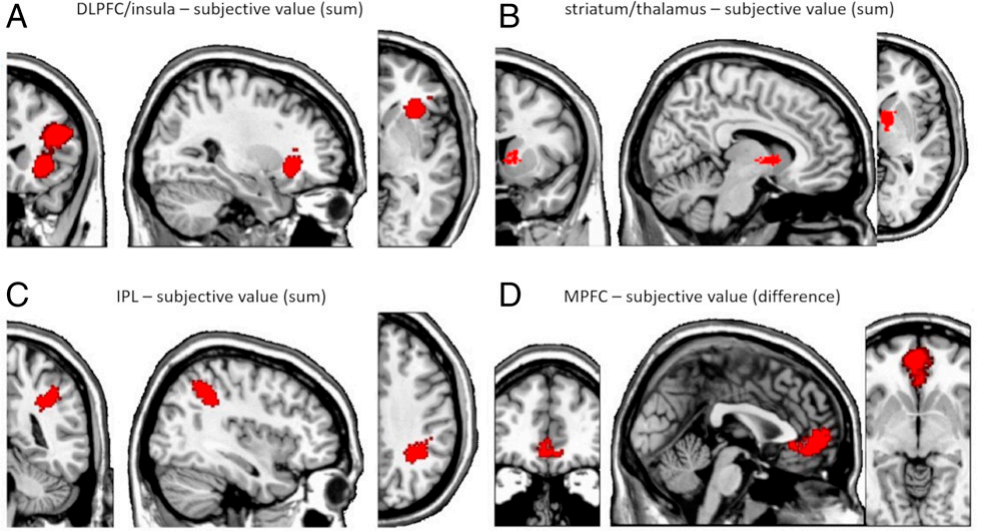
Subjective value coding common to EU, PT, and MVS models. (*A*–*C*) Subjective state value signal (sum of subjective values of the two options) correlated with activity in (*A*) right dlPFC and insula, (*B*) striatum, and (*C*) right IPL from lottery presentation to time of decision. (*D*) At the time of decision, subjective value differences correlated with activity in vmPFC. The cluster-inducing voxel-level threshold was *P* < 0.001 and *P* < 0.05 family-wise error corrected at the cluster level in each contrast separately, followed by inclusive masking across the three models. The respective figures were masked to show only one cluster per figure for visual clarity.

We used an inclusive masking approach to test whether the activity of some regions correlated with the subjective state value computed by each model in the GLMs at the time of option presentation. We then calculated the intersection image of all three models using whole-brain cluster-level family-wise error–corrected images for each model [*P* < 0.05 cluster-level family-wise error corrected with a cluster inducing voxel-level threshold of *P* < 0.001, uncorrected, and a minimum cluster size of 165 voxels (27); for peak activations for individual models, see *SI Appendix*, Table S1]. This procedure identified activity in the right dorsolateral prefrontal cortex (dlPFC) and bilateral insula (Fig. 3*A*), the striatum extending into the thalamus (Fig. 3*B*), and the right lateral intraparietal lobule (IPL; Fig. 3*C*). Thus, these regions all encode subjective state value at option presentation regardless of the behavioral model used to compute subjective value, demonstrating that different models with similar behavioral predictions result in similar brain activations reflecting subjective value.

The same conclusion but with different brain regions emerged for subjective value differences at the time of choice in the second GLMs. Again, using the inclusive masking procedure, we found a large cluster common to all models in the medial orbitofrontal and the anterior cingulate cortex, which together we denote as ventromedial prefrontal cortex (vmPFC; Fig. 3*D*, *P* < 0.05 whole-brain cluster-level corrected). Thus, activity in these regions correlated with the subjective value differences computed from the EU, PT and MVS models. Together, the data reinforce the strong similarity between models and reveal that neural encoding of subjective value in these regions is model invariant if traditional parametric methods are used. This finding highlights the notion that model comparison at the behavioral level alone could lead to unjustified conclusions that the best-fitting behavioral model is being implemented at the neural level. However, the finding of commonly identified brain regions also provides cause for relief, in the sense that previous studies using different approaches correctly identified the same regions (28).

Next, we aimed to determine the brain mechanisms of subjective value computation in these commonly identified brain regions by formal model comparison at the neural level. In particular, we employed Bayesian analysis methods to quantify the evidence favoring each of the three models. To do this, we constructed functional regions of interest (ROIs) from the intersecting subjective state value regions (i.e., striatum, dlPFC, insula, and IPL) and intersecting subjective value difference regions (i.e., vmPFC). We then performed one first-level Bayesian analysis for each model and ROI in each participant and passed the results to a Bayesian model comparison (29, 30). For this analysis, we computed the expected model frequencies (the probability that the model generated the data for any randomly selected subject) and used these to compute exceedance probabilities (i.e., the confidence in the model having the highest expected frequency relative to all tested models) with the Variational Bayesian Analysis (VBA) toolbox (31). Strikingly, we found that different models best captured subjective state value computations in the different regions.

**Fig. 4.**
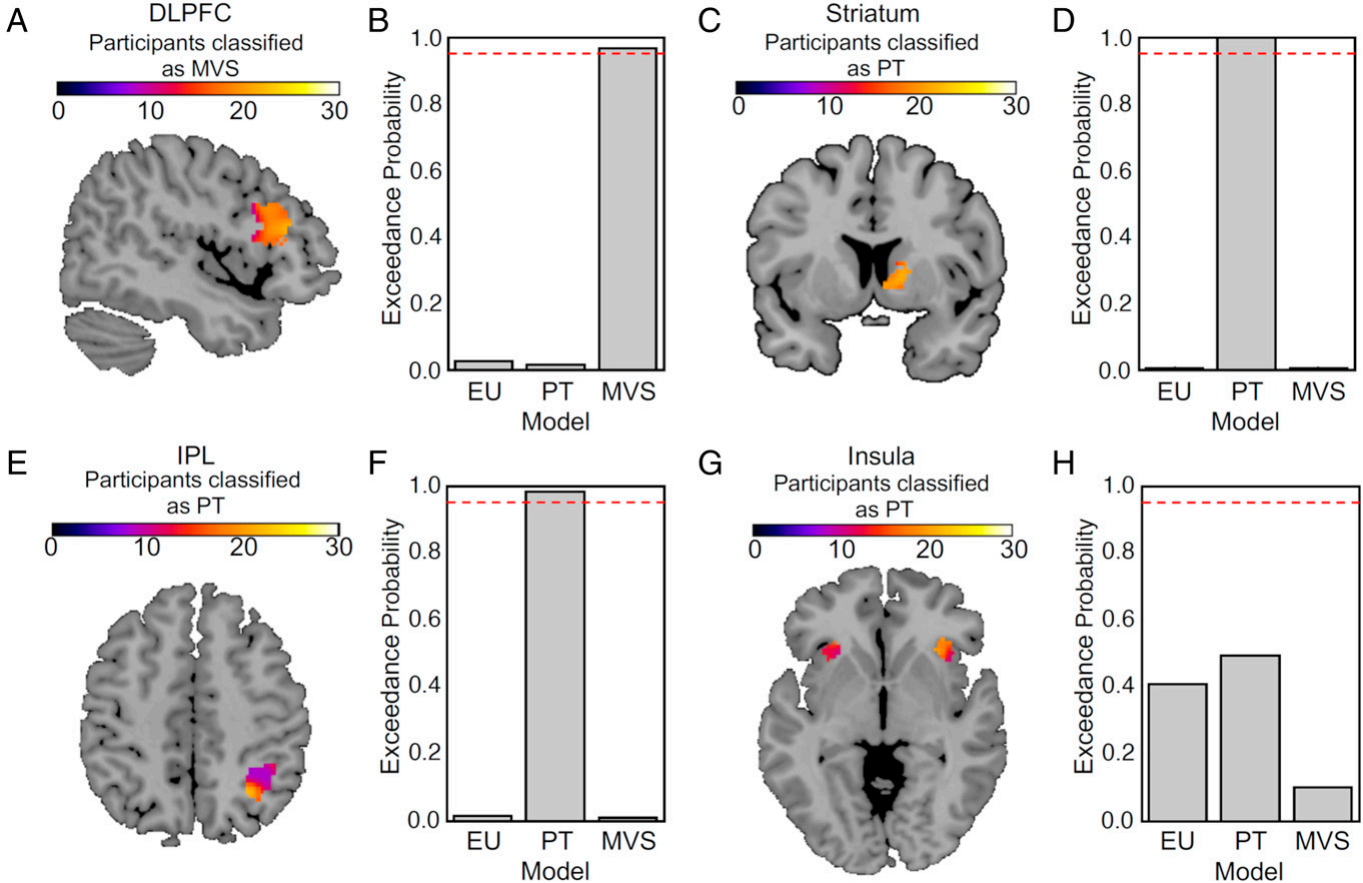
Neural Bayesian model comparison in regions encoding subjective state (sum) value. Colors correspond to the number of participants where a model explained the neural data better than the other two models (not the strength of the blood oxygen level–dependent signal). Red dashed lines in the bar plots indicate an exceedance probability of 0.95. (*A* and *B*) The MVS model explained neural data better than the PT or EU model in the right dlPFC, with an exceedance probability (the belief that one model is better than the other two models at explaining the underlying neural activity) of 0.96. In the striatum (*C* and *D*) and IPL (*E* and *F*), PT explained the encoding of subjective value better than the MVS and EU models, with exceedance probabilities of 1.00 and 0.98, respectively. (*G* and *H*) Neural activity in the insula could not be explained categorically by any of the three models.

Specifically, activity in the dlPFC related to state value (i.e., the summed subjective values of the two presented options) was best explained by the MVS model (Fig. 4*A*), with an exceedance probability (i.e., the belief that this model explains the underlying neural activity better than the other two models) of 0.96 (Fig. 4*B*). By contrast, the striatal and IPL activations were best explained by the PT model (Fig. 4 *C*–*F*), with exceedance probabilities of 1.00 and 0.98, respectively. Exploratory analyses also suggested that PT-conforming behavior is associated with weaker connectivity between IPL and other value-processing regions, particularly vmPFC (*SI Appendix*, *Supplementary Analyses*). Activity in the insula could not be satisfactorily classified by any of the three models (Fig. 4*G*). Taken together, the data suggest a surprising dissociation between the dlPFC and the IPL/striatum during subjective state value encoding. Given the characteristic theoretical elements of the two models, dlPFC appears to encode subjective value primarily in the form of summary statistics entailed by a lottery whereas IPL and striatum do so through weighting subjective values of outcomes with their distorted probabilities.

**Fig. 5.**
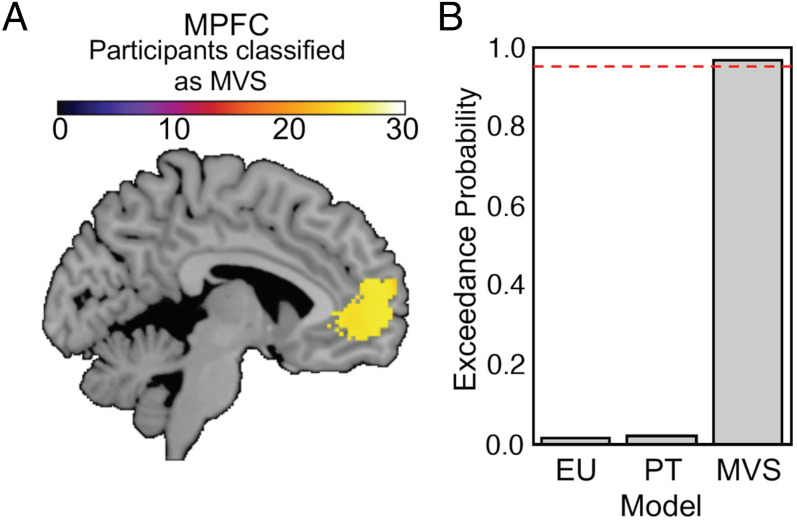
Neural Bayesian model comparison in regions encoding subjective value difference at the time of the decision. (*A*) The MVS model best explained neural activity in the vmPFC with an exceedance probability of 0.96 (*B*).

We also assessed subjective value difference regions [i.e., vmPFC activity at the time of the decision (Fig. 5*A*)]. We found that in the vmPFC, the MVS model significantly outperformed the PT and EU models. The exceedance probability for MVS was 0.96 (Fig. 5*B*). Together, these findings provide compelling evidence that the model-invariant value-processing regions in frontal cortical areas encode subjective value as proposed by the summary statistics approach, while subcortical and parietal areas encode value according to PT.

Finally, to confirm that model comparison at the neural level indeed provides more insights than the standard model-based functional MRI (fMRI) approach, we performed a formal model comparison at the behavioral level. Using the log likelihoods for the best-fitting EU, PT, and MVS models for each participant, we computed the small-sample–corrected Akaike Information Criterion (AICc) for each model fit (thus compensating for the different numbers of free parameters in each model). AICc values were then formally compared using the VBA toolbox (31) to compute the expected model frequencies and exceedance probability for each model. Bayesian model comparison showed that at the group level, participants’ choices were best explained by the PT model with an exceedance probability of 0.99 (although there was interindividual variation in model evidence, see *SI Appendix*, Fig. S1). Thus, using a more traditional approach of assessing behavioral model fits first (which is problematic when models make similar predictions) and taking solely the winning model to the neural level, we would have prematurely concluded that all the subjective value–coding regions identified were implementing PT.

## Discussion

Our results indicate that model comparison at the neural level can provide a way to distinguish between models that make similar behavioral predictions. The MVS model, typically utilized to explain choice preferences in behavioral ecology and finance, best captured the neural computation of subjective value in ventromedial and dorsolateral areas of the prefrontal cortex. By contrast, in the striatum and parietal cortex, subjective value computation best reflected PT. More generally, although all models commonly identified brain regions that have been associated with encoding subjective value under a traditional model-based fMRI approach, Bayesian model comparison at the neural level revealed that the three models differentially explain how these regions compute subjective value. In these brain regions, the PT and MVS models appear to explain neural coding of subjective value better than EU theory.

The finding that models with similar behavioral predictions differentially explain neural activity encoding subjective value reinforces Marr’s (32) view that a multilevel approach is needed when we aim to understand a cognitive function. The need to perform model comparisons at the neural level can be exacerbated when neural data are insensitive to large changes in model parameters obtained by behavioral fits (33). Although we investigated the neural mechanisms encoding subjective value during risky decisions of humans, there is reason to believe that the standard approach of model-based neuroscience could also misrepresent, or at least underspecify, neural computations in other domains and species. Likely candidates include related domains of subjective valuation [e.g., competing models of temporal discounting of delayed choice options (e.g., ref. 34)] and entirely different domains such as recognition memory (35) or mental processing modes (36). Moreover, this approach is not limited to human neuroscience but might also be applied to data from animal models of cognitive processes [e.g., comparing different models of decisions under risk (37) or with delayed outcomes (38)]. In all of these domains, Bayesian model selection (BMS) at the neural level could supersede the standard approach of fitting only the best behavioral model to neural data and thereby provide insights about brain function. Of course, model comparisons with neural data can be just as inconclusive or ambiguous as with behavioral data. Therefore, this approach should not be understood as the only solution to the problem of correlated behavioral models. Rather, model comparisons on the neural level may provide a more realistic perspective on the biological implementation of the choice process, in-keeping with Marr’s framework.

There are several potential explanations for why MVS and PT described neural activity better than EU. Under MVS, the values of uncertain prospects are calculated using individual attitudes toward the statistical moments of the underlying reward distribution. This is computationally more efficient than EU or PT because preferences can be represented by additive combination of simple characteristics of distributions (39). Mean-variance approaches have been shown to optimize the allocation of risky assets in portfolio selection theory and explain broader classes of preferences than PT (40), suggesting that they are more resistant to market forces in the world of financial investment. It is conceivable that neural systems facing uncertainty may have faced similar challenges in their evolutionary history. Another advantage of the summary statistics approach is that it facilitates learning by allowing the decision maker to compute the mean, variance, and skewness of a lottery through repeated experience of outcomes, with these estimates converging to the true moments of the distribution through the law of large numbers (41). Thus, dorsolateral and ventromedial prefrontal regions may facilitate efficient and experience-dependent representations of subjective value and may be involved in learning the statistical moments of the value and probability distributions of the offered lotteries. In contrast, PT allows for more precise and experience-independent representation of subjective value (39). It is therefore conceivable that PT representations are particularly important when learning is not (yet) possible and when subjective valuation is based on heuristic appraisals of numerically displayed options. Our findings suggest that these functions are underpinned by the striatum and parietal cortex.

A central feature of PT is probability distortion, which has been reported in the striatum (42). This is in line with our finding of PT best capturing striatal computation of subjective state value. One prediction of the MVS approach is that the summary statistics of reward distributions are represented separately at the neural level. This indeed appears to be the case with previous studies showing mean (expected value) coding in the lateral PFC (43) and variance coding in the insula, anterior cingulate, lateral orbitofrontal cortex, and posterior parietal cortex (13, 43–45). While skewness appears to be represented also in the insula and ventral striatum (13, 45–47), it remains to be seen whether single neurons encode skewness together with other moments. In the orbitofrontal cortex, single neurons seem to code moments separately, at least with regard to the mean and variance of reward distributions (48). The separate neural representation of summary statistic terms together with our finding of preferential fit of prefrontal activity by MVS may suggest that evolution favored the relatively simple solution to implementing subjective value computation of MVS for context-sensitive regions that keep track of the overall reward distributions in the longer term.

Our study suggests that the brain represents subjective value in multiple forms. This finding may help explain how the description-experience gap arises, according to which people show PT-like behavior when outcomes and probabilities are described (as in our paradigm) but not when they are experienced (49). Specifically, with decisions from description, decision makers typically overweigh the probability of rare events in line with PT and with PT-like representations. In contrast, when the outcomes of decisions are experienced through repeated sampling, decision makers show no overweighing or even underweighting of rare events (49), in line with EU-like or other representations. Furthermore, a recent study using theoretical models and data-driven machine learning algorithms has shown that behavior in a wide variety of risky choice problems can be explained well by a combination of EU- and PT-like functions, relying more on one or the other model depending on the specific choice problem (50). Although speculative, our findings could suggest a viable neurobiological implementation of such mixture models within the domain of decisions from description.

The present approach is not limited to value-based decision-making. Our procedure is applicable in any area of research in which competing models make similar predictions on the behavioral level (e.g., learning and memory). However, there are also limitations to this procedure. Exceedance probability specifies well the strength of belief that given data has been generated by the models in the set, but it only makes inferences about the models in this set (30, 31). We cannot conclude that the model preferred by the Bayesian model comparison was indeed the data-generating model, only that this model explains the data best among all the models in the set. Moreover, given the strong overlap of behavioral predictions across models (Fig. 2), future research may want to design experimental tasks that more strongly dissociate the different models of value-based decision-making (e.g., by tailoring the lotteries to each participant individually based on a screening session).

In conclusion, we have revealed how the brain computes subjective value by performing model comparisons also on the neural level instead of limiting them to the behavioral level and only taking the winning model to the neural level. Our findings suggest that different brain regions implement different models of subjective value computation. Naturally, this proposition will benefit from boundary and generality tests inside and outside of decision-making. It will be particularly interesting to study in which context one rather than another model-specific brain region drives behavior. Importantly, our finding that different brain regions implement different computational models has the potential to contribute to a more sophisticated understanding of the neurobiological underpinnings of value-based decision-making.

## Experimental Procedures

### Participants.

All 31 participants (20 females) were healthy, right-handed, had normal or corrected-to-normal vision, and reported no history of psychiatric disorders. The first three participants were pilot participants, saw only 135 of the 180 trials used in the main study, and are therefore excluded from analysis. One further participant was excluded due to a scanner crash during one of the functional runs. Thus, the final sample consisted of 27 participants (17 females, age: M = 28.3 y, SD = 5.0 y). The study was approved by the Cantonal Ethics Commission of Zurich. All participants gave informed consent prior to participating in the fMRI task.

### Experimental Task.

Participants completed a risky decision-making task in the MRI scanner. At the beginning of each trial, two lotteries were displayed on the screen in the form of pie charts; participants had to decide which lottery they preferred. For each lottery, there were two possible monetary outcomes, and the shading of segments next to each outcome indicated the probability associated with that magnitude (with each segment indicating a 10% chance). A list of the lotteries used in the experiment is provided in *SI Appendix*, Tables S2 and S3. Lottery pairs were explicitly constructed to differentiate between the three models used in the experiment and were selected from two previous studies (1, 7). Note that it is extremely hard to design tasks that clearly dissociate between models with similar predictions. Indeed, even though the present lotteries have been designed (7) with the explicit aim of differentiating between expected utility and cumulative PT (assuming a reference point of zero), the model predictions still correlate strongly (Fig. 2). It therefore becomes necessary to use a statistical approach that quantifies the evidence for each of the models per participant and over the group of participants (see *Behavioral Models* and* BMS for fMRI data*). The point of this paper is not to demonstrate that our paradigm is superior to others in distinguishing between the models of value-based decision-making but to show a way of modeling behavioral and neuroimaging data to enhance the insights generated from any of these experimental paradigms.

Following Bruhin and colleagues (7), our lotteries had a wide range of magnitudes (x∈{0, 5, 7, 10, 12, 13, 15, 16, 17,18, 19, 20, 22, 25, 27, 30, 33, 35, 36, 38, 40, 42, 43, 47, 50}) and probabilities (p∈{0, 0.05, 0.1, 0.2, 0.25, 0.3, 0.35, 0.4,0.5, 0.6, 0.7, 0.75, 0.8, 0.85, 0.9, 0.95, 1}), allowing for probability distortion as well as varying variance and skewness to assess MVS preferences. Participants were instructed to make their decision by pressing the left or right button on a button box and allowed up to 5 s to make a decision. Following their decision, feedback indicating the selected option (or failure to make a choice) was displayed until 5 s after lottery pair onset (but to prevent learning, the lotteries were never actually played out during the experiment). There were 180 decision trials evenly spaced among three functional runs. An additional 45 trials consisting of a prolonged fixation cross were interspersed between lottery trials and lasted for 5.5 s. These null trials served to increase design efficiency (51). The experimental task was programmed using the Cogent 2000 toolbox (version 1.32) and Matlab R2010b (The MathWorks Inc.).

After participants completed the task in the scanner, four trials were selected for payment and implemented outside the scanner. Participants earned points, which were converted to Swiss Francs (CHF) at the end of the experiment with 1 point = CHF 0.25. The four trials selected for payment were determined by a computerized random number generator. The lottery chosen by the participant on each of the four determined trials was then played out for real money. If the chosen lottery had more than one possible outcome, participants rolled a 20-sided die to play the lotteries, with each number being multiplied by 0.05 to implement probability. For example, if the lottery gave a 20% chance of winning 0 points and an 80% chance of winning 35 points, then a roll of 1 to 4 resulted in winning 0 points and a roll of 5 to 20 resulted in winning 35 points. Participants earned CHF 30.63 on average during the behavioral task in addition to a fixed payment of CHF 25 for participating in the experiment.

### Behavioral Models.

For all trials, we characterized the presented lotteries and fitted models as follows. We denoted a lottery by *x* = (*x1*, *p*; *x2*, 1-*p*), where *x1* > *x2* and *x* gave outcome *x1* with probability *p* and outcome *x2* with probability 1-*p*. On a small number of trials, one of the lotteries offered an amount with certainty (*p* = 1). For each participant, we estimated the parameters of EU, PT, and MVS using the following model specifications:

EU model:

[1] $$U_{EU}\left(x\right)=p{\left(x1\right)}^{\rho }+\left(1-p\right){\left(x2\right)}^{\rho },$$

where ρ describes the curvature of the value function.

PT model:

[2] $${\text{U}}_{\text{PT}}\left(\text{x}\right)=\text{w}\left(\text{p}\right){\left(\text{x}1\right)}^{\text{ρ}}+\left(1-\text{w}\left(\text{p}\right)\right){\left(\text{x}2\right)}^{\text{ρ}},$$

where ρ describes the curvature of the value function, and

[3] $$\text{w}\left(\text{p}\right)=\frac{{\text{δp}}^{\text{γ}}}{{\text{δp}}^{\text{γ}}+{\left(1-\text{p}\right)}^{\text{γ}}}$$

denotes probability distortion according to the Goldstein–Einhorn function (52), with δ and γ corresponding to the elevation and curvature of the probability distortion function, respectively. As all outcomes were nonnegative, we omitted the loss domain from our PT model.

MVS model:

[4] $${\text{U}}_{\text{MVS}}\left(\text{x}\right)={\text{β}}_{\text{μ}}\left(\text{Mean}\left(\text{x}\right)\right)+{\text{β}}_{\text{σ}}(\text{Variance}\left(\text{x}\right))+{\text{β}}_{\text{γ}}(\text{Skewness}\left(\text{x}\right)),$$

where $\beta_{\mu }$, $\beta_{\sigma }$, and $\beta_{\gamma }$ describe a participant’s preferences for the mean, variance, and skewness of the lottery, respectively. For a lottery with a sure outcome (*p* = 1), skewness is technically undefined. For simplicity, we assigned a skewness(x) of 0 to these lotteries.

We estimated all three models using maximum likelihood and therefore needed to adopt a stochastic choice rule for use as the likelihood function. We adopted the Luce choice rule, which is commonly used for probabilistic choice. The Luce choice rule, equivalent to a softmax function with the temperature parameter equal to 1, assumes that choice mistakes are most likely to occur when they are not very costly (i.e., the values of the two options are close). Let $V_{L}$ and $V_{R}$ indicate the subjective values of the left and right lotteries, respectively, on a given trial, and let $P_{L}$ be the probability of choosing the lottery on the left. Then, the probability of choosing the left option corresponds to:

[5] $${\text{P}}_{\text{L}}=\frac{1}{1+\text{exp}({\text{V}}_{\text{R}}-{\text{V}}_{\text{L}})}.$$

Using this choice rule, we obtained the log-likelihood function,

[6] $$\text{LL}={\text{Σ}}_{\text{t}}[{\text{I}}_{\text{L}}\left({\text{P}}_{\text{L}}\right)+\left(1-{\text{I}}_{\text{L}}\right)\left({\text{P}}_{\text{R}}\right)],$$

where t represents a trial, and $I_{L}$ is an indicator function equal to 1 when the lottery on the left was selected and 0 otherwise. We then obtained the best-fitting parameter estimates by maximizing the log-likelihood separately for each model using the Nelder–Mead method (53). Behavioral models were estimated using R statistical software (54). For the EU and PT models, theoretical considerations and empirical evidence suggest that plausible parameter values will be positive (55, 56). Therefore, we constrained the parameters to be positive by using the transformed variable $e^{\text{ln}(\psi )}$ for each parameter ψ. For the MVS model, we assumed that participants prefer increasing mean values ($\beta_{\mu }>0)\;$ but remained agnostic regarding the sign of the variance and skewness parameters. The sign of the variance parameter is often interpreted as a measure of risk preference and could be negative or positive depending on a participant’s risk attitude ($\beta_{\sigma }<0$ implies risk aversion, $\beta_{\sigma }>0$ implies risk tolerance). At present, there is a lack of evidence regarding skewness preferences and therefore plausible values for $\beta_{\gamma }$ (12, 14–16). Since optimization procedures can converge to local maxima/minima, which do not necessarily correspond to the global maximum/minimum, we used several sets of starting values for parameters. Log-likelihood was used to assess model fits, and the small-sample corrected AICc was used to classify participants into behavioral types and perform model comparison at the group level. Following conventional rules-of-thumb for model classification (57), participants were classified as a PT-type if 1) $\mathrm{AIC}c_{EU}-\mathrm{AIC}c_{PT}>2$ and 2) $\mathrm{AIC}c_{\mathrm{MVS}}-\mathrm{AIC}c_{PT}>2$. Formal model comparison was performed by passing the AICc values to the group Bayesian Model Comparison function of the VBA toolbox (31). Finally, we generated choice predictions by simulating the choices for each model, using the optimized parameters from the fitting procedure and by selecting the option with the highest choice probability (i.e., predict Left option if $P_{L}>P_{R}$ and vice versa). Using the best-fitting parameters for each participant and model, we computed the subjective (cardinal) values of the options used in the experiment using Eqs. **1**, **2**, and **4**. Ordinal ranks were calculated by sorting the cardinal values for each participant and model from minimum to maximum.

#### fMRI data collection.

Data were acquired with a 3T Philips Achieva scanner. Functional images were taken with a T2-weighted gradient echo-planar imaging sequence (repetition time [TR] = 2,500 ms, echo time [TE] = 36 ms, and flip angle = 90°). Whole-brain coverage was achieved by 37 slices, acquired in ascending order (3-mm thickness, 0.5-mm gap, and in-plane voxel size 2 × 2 mm). Participants' heads were restrained with foam pads to decrease head movement. Functional imaging data were acquired in three separate sessions, each lasting about 10 min. High-resolution structural T1-weighted three dimensional–turbo field echo anatomical scans of the whole brain were also acquired for each participant (voxel size 1.1 × 1.1 × 0.6 mm, TR = 7.5ms, TE = 3.5 ms, and flip angle = 8°).

#### fMRI data analysis.

We used a standard rapid-event–related fMRI approach in which evoked hemodynamic responses to each event type are estimated separately by convolving a canonical hemodynamic response function (HRF) with the onsets for each event and regressing these against the measured fMRI signal. Using statistical parametric mapping (SPM12; Functional Imaging Laboratory, University College London) we performed slice timing correction, spatial realignment, normalization to the standard echo-planar imaging template and spatial smoothing using an isometric Gaussian kernel with a full-width at half-maximum (FWHM) of 10 mm.

To investigate the neural encoding of subjective values for each model, we constructed a GLM using model-based subjective values as parametric modulators. This GLM included a regressor for the trial onset with a duration until the time the participant entered the decision. This onset regressor was parametrically modulated on a trial-by-trial basis by the sum of the subjective values of the two lotteries on screen (state value), either for the EU model, the PT model, or the MVS model. A second regressor modeled the time of the decision. This onset regressor was parametrically modulated on a trial-by-trial basis by the difference in the subjective value of the lotteries on screen (subjective value difference), either for the EU model, the PT model, or the MVS model. Missed trials, in which a decision was not entered within 5 s, and null trials were each modeled as separate conditions (regressors of no interest) in the design matrix. Thus, the GLM included the following regressors:

0) Constant;
1) Onset of valid trials—time when the lotteries were presented in the trials in which the participant made a choice in time (regressor duration until response);
2) State value—parametric modulator of (1), using the trial-specific sum of subjective values of both lotteries (Eqs. **1**, **2**, or**4**);
3) Decision—time when the participant conveyed their decision, that is, indication of the selected option (regressor duration 0 s);
4) Value difference—parametric modulator of (3), using the trial-specific difference between subjective values of lotteries (Eqs. **1**, **2**, or**4**);
5) Onset of null trials (regressor duration 0 s);
6) Onset of missed trials—time when the lotteries were presented in the trials in which the participant did not make a choice in time (regressor duration 5s);
7) State value of error trials—parametric modulator of (6), using the trial-specific sum of subjective values of both lotteries;
8) Error term.

The regressors 0 to 7 are repeated twice because the experiment was performed in three runs with short breaks in between. All regressors were convolved with the canonical HRF, including time and dispersion derivatives to account for differences in latency and shape of the responses. We built one GLM for each of the three theoretical models of value computation (EU, PT, and MVS). To ease the comparability of subjective values between the three models, we used the certainty equivalents of the respective utility value (p. 186 of ref. 8).

The three GLMs enabled us to investigate the neural encoding of subjective value based on the assumption that the subjective values would be processed in the brain at the time of option presentation/choice consideration (state value) and the time of the decision itself [difference value (58–60)]. We weighted the regression coefficients from the two parametric modulators (i.e., the sum of the subjective values of both options during the lottery presentation and the difference in subjective values between both options at the time of the response) with a 1 at the individual participant level and took the resulting contrast images to group-level Student’s *t* tests. These second-level models identified regions coding subjective value according to the different models. By using inclusive masking of activations commonly identified by all three models, we determined regions that compute subjective value in a model-invariant manner. From the resulting intersection image, clusters were identified and ROIs for subsequent BMS created using xjview and MRIcron packages.

#### BMS for fMRI data.

To test whether the subjective values generated by one particular model explain neural computation of subjective values substantially better than the alternative models, we performed BMS. Specifically, we compared the log-evidence maps from alternative models separately for state value and value differences. This allowed us to identify the model which best explained neural activity related to subjective value computation.

We implemented BMS in SPM12 by following previously described procedures (29). We repeated the same preprocessing as described in *fMRI data analysis* but omitted smoothing, as the log-evidence maps produced by the first-level analysis are smoothed before conducting BMS. Our BMS analysis assessed the three models (EU, PT, and MVS) in the common subjective value regions (dlPFC, striatum, insula, and IPL for state value, vmPFC for difference value). Therefore, we compared the model fits not in the whole brain but only in the areas that the preceding analyses associated with state value or value difference for all three tested models.

In the first-level Bayesian analyses, we included either the sum of subjective values (state value) and the absolute difference of subjective values (difference value) as a parametric modulator. The neural models were estimated three times, once for each subjective value model, using model- and participant-specific subjective values from EU, PT, or MVS. Log-evidence maps resulting from the first-level analysis were smoothed using an 8-mm FWHM Gaussian kernel. We then conducted a voxel-wise random effects BMS analysis in the ROIs. Bayesian model comparison was performed at the second level for each model (30). This allowed us to calculate the expected posterior probabilities for the EU, PT, and MVS models along with the exceedance probability (quantifying the belief that one model is more likely than the other). For model comparison at the ROI level, we averaged the log-evidence values within each ROI for each participant and passed these to the VBA toolbox (31).

## Supplementary Material

Supplementary File

## Data Availability

Anonymized statistic parametric maps data have been deposited in Neurovault (https://identifiers.org/neurovault.collection:9604).
