# Supplementary material for: Testing models at the neural level reveals how the brain computes subjective value
Source: Proc Natl Acad Sci U S A. 2021 Oct 22;118(43):e2106237118. doi: 10.1073/pnas.2106237118 (PMC8639327; doi:10.1073/pnas.2106237118)
Supplement: Supplementary File [file pnas.2106237118.sapp.pdf]

SUPPLEMENTARY MATERIAL

Testing models at the neural level reveals how the brain computes subjective value

Tony B. Williams[1]*, Christopher J. Burke[1]*, Stephan Nebe[1]*, Kerstin Preuschoff[1,2], Ernst Fehr[1], Philippe N. Tobler[1]

*these authors contributed equally

Supplementary background: State value vs. value difference

We use state value during option presentation and value difference at choice to analyze the neural data in the main paper. While we determine state value as the sum of the subjective values of the options, it could in principle serve as input for decisions when options are presented alone and decision makers decide whether to continue to do what they do in a given state. Accordingly, state value is relevant to anybody interested in value-based choice. Moreover, it is well known that the brain represents state value also in choice situations (e.g. 1), in-keeping with the notion that multiple value signals are represented in the brain.

In contrast to state value, the value difference between options is a classic decision value signal that correlates strongly with the probability of choosing one option over the other (e.g. 2) and is commonly used in decision neuroscience (e.g. 3–6). Note that the larger the value difference, the more evidence there is in favor of one over the other option. Accordingly, computational decision models directly transform value difference into the probability of choosing one action via a softmax function (e.g. 7). It is worth noting that choice difficulty (e.g. 8) can be defined as the inverse of the value difference. However, choice difficulty tends to activate different regions than those coding value difference (see results; (5, 6)). Of course, the sum and difference of subjective option values are not the only important variables related to value-based decision making. Yet, because they are defined independently of behavior, they are less subject to bias (arising e.g., from individual differences) and confound than choice-dependent value measures (e.g. 5, 9) and as such useful and informative.

Supplementary tables and figure

Table S1. BOLD responses from parametric analysis over the whole group of participants (N=27) for each model (Expected Utility, Prospect Theory, Mean-Variance-Skewness) for the sum of subjective values for both presented options during option presentation and difference between subjective values of options at decision time ($p_{uncorr.}$<.001, k=20; areas named with the Anatomy Toolbox, version 2.2b, (10–12))

Peak MNI coordinates x	y	z	Peak Z-value	Hemisphere	Anatomical region
Expected utility model; Subjective value sum					
48	2	18	5.49	R	Inferior frontal gyrus (cluster extends into caudate)
32	-48	34	4.69	R	Intraparietal sulcus
34	-90	-8	4.49	R	Inferior occipital gyrus
-26	24	-4	4.48	L	Insula
-50	28	24	4.39	L	Inferior frontal gyrus
-32	-88	-8	4.38	L	Inferior occipital gyrus
-24	-62	42	4.35	L	Superior parietal lobule
36	-54	-16	4.08	L	Fusiform gyrus
14	22	42	4.02	R	Superior medial gyrus
-4	-74	-26	3.84	L	Cerebellum
26	10	54	3.79	R	Superior frontal gyrus
-36	54	-2	3.75	L	Middle orbital gyrus
-36	0	30	3.70	L	Precentral gyrus
-2	-82	-4	3.66	L	Calcarine gyrus (V1)
-38	-50	-16	3.53	L	Fusiform gyrus
8	-62	42	3.41	R	Precuneus
Expected utility model; Subjective value difference					
-28	-14	-22	4.86	L	Hippocampus
8	48	-6	4.61	R	Middle orbital gyrus
10	-6	28	4.45	R	Midcingulate cortex
66	-12	-8	4.29	R	Superior temporal gyrus
-26	20	10	3.83	L	Insula
16	-24	52	3.78	R	Posterior medial frontal cortex
-12	2	-12	3.59	L	Olfactory cortex
36	-52	12	3.57	R	White matter
-12	-46	-28	3.48	L	Cerebellum
Prospect theory model; Subjective value sum					
46	10	18	5.24	R	Inferior frontal gyrus (cluster extends into caudate)
-28	26	-6	4.49	L	Insula
34	-50	38	4.38	R	Angular gyrus
-48	26	22	4.33	L	Inferior frontal gyrus
34	-90	-8	4.15	R	Inferior occipital gyrus
14	22	42	4.07	R	Superior medial gyrus
-32	-88	-8	4.02	L	Inferior occipital gyrus
-40	54	0	4.00	L	Middle frontal gyrus
-12	12	-2	3.94	L	Caudate*
36	-54	-16	3.90	R	Fusiform gyrus
-24	-62	42	3.84	L	Superior parietal lobule
-34	2	30	3.83	L	Precentral gyrus
-2	-78	-6	3.67	L	Lingual gyrus (V1)
-4	-74	-26	3.66	L	Cerebellum
4	-54	-16	3.51	R	Cerebellar vermis

10	-60	42	3.40	R	Precuneus
-26	-76	24	3.40	L	Middle occipital gyrus
18	-52	-28	3.37	R	Cerebellum
Prospect theory model; Subjective value difference					
-8	52	-8	5.34	L	Middle orbital gyrus
-30	-32	-12	4.61	L	Hippocampus
-14	-50	26	4.58	L	Precuneus
-12	-12	52	4.42	L	Midcingulate cortex
-50	-18	50	4.12	L	Postcentral gyrus
-20	-46	70	4.02	L	Superior parietal lobule
24	-12	-22	3.96	R	Hippocampus
-22	28	40	3.80	L	Middle frontal gyrus
38	-56	16	3.78	R	Middle temporal gyrus
-48	0	10	3.71	L	Rolandic operculum
-54	-64	16	3.56	L	Middle temporal gyrus
52	-18	-16	3.51	R	Middle temporal gyrus
66	-26	4	3.44	R	Superior temporal gyrus
Mean-variance-skewness model; Subjective value sum					
34	22	-4	4.73	R	Insula (cluster extends into inferior frontal gyrus)
-28	22	-4	4.37	L	Insula
-10	-16	-12	4.17	L	Thalamus
26	-42	40	3.78	R	Intraparietal sulcus
14	6	0	3.67	R	Pallidum (cluster extends into caudate)
18	-52	-28	3.64	R	Cerebellum
-12	6	-2	3.63	L	Pallidum (cluster extends into putamen)
32	-46	-20	3.63	R	Fusiform gyrus
-36	2	30	3.56	L	Precentral gyrus
34	46	14	3.51	R	Middle frontal gyrus
10	22	38	3.46	R	Midcingulate cortex
-2	-82	-4	3.42	L	Calcarine gyrus (V1)
Mean-variance-skewness model; Subjective value difference					
8	30	4	4.37	R	Superior medial gyrus (cluster extends into middle orbital gyrus and anterior cingulate cortex)
20	-56	32	4.08	R	Precuneus
-38	-32	-10	4.03	L	Hippocampus
36	-18	-18	3.99	R	Hippocampus
2	-12	32	3.94	R	Midcingulate cortex
-10	-52	18	3.92	L	Precuneus
-58	-6	-12	3.91	L	Middle temporal gyrus
44	-34	-6	3.83	R	Hippocampus
56	-14	-12	3.77	R	Middle temporal gyrus
-30	-6	-16	3.64	L	Hippocampus
-30	-40	66	3.52	L	Postcentral gyrus
-46	-62	8	3.51	L	Middle temporal gyrus
36	46	24	3.49	R	Middle frontal gyrus

* No classification provided by Anatomy Toolbox, instead named using the Automated Anatomical Labelling Atlas (13).

Table S2. Lottery pairs (1-90) used in the study.

	Left Lottery			Right Lottery				Left Lottery			Right Lottery		
Pair	p	x_1	x_2	p	x_1	x_2	Pair	p	x_1	x_2	p	x_1	x_2
1	0.50	20	0	0.30	25	0	46	0.25	20	10	0.15	45	5
2	0.50	20	0	0.30	30	0	47	0.25	20	10	0.15	40	5
3	0.50	20	0	0.95	10	0	48	0.25	20	10	0.05	45	10
4	0.50	20	0	0.20	30	0	49	0.75	20	10	0.60	20	15
5	0.50	20	0	0.20	35	0	50	0.75	20	10	0.60	30	5
6	0.50	20	0	0.20	40	0	51	0.75	20	10	0.50	25	10
7	0.10	40	0	0.20	35	0	52	0.75	20	10	0.50	30	5
8	0.10	40	0	0.20	30	0	53	0.75	20	10	0.90	20	5
9	0.10	40	0	0.20	25	0	54	0.75	20	10	0.90	25	0
10	0.10	40	0	0.40	15	0	55	0.95	40	10	0.75	40	25
11	0.10	40	0	0.40	20	0	56	0.95	40	10	0.75	35	25
12	0.10	40	0	0.40	25	0	57	0.95	40	10	0.60	50	20
13	0.90	40	0	0.70	50	10	58	0.95	40	10	0.60	45	20
14	0.90	40	0	0.70	40	10	59	0.95	40	10	0.50	45	25
15	0.90	40	0	0.70	40	15	60	0.95	40	10	0.50	35	30
16	0.90	40	0	0.50	40	20	61	0.05	50	20	0.20	40	20
17	0.90	40	0	0.50	40	15	62	0.05	50	20	0.20	35	20
18	0.90	40	0	0.50	35	20	63	0.05	50	20	0.30	30	20
19	0.10	20	10	0.25	15	10	64	0.05	50	20	0.30	35	20
20	0.10	20	10	0.25	30	5	65	0.05	50	20	0.50	25	20
21	0.10	20	10	0.25	25	5	66	0.05	50	20	0.50	30	20
22	0.10	20	10	0.35	15	10	67	0.25	20	10	0.40	25	5
23	0.10	20	10	0.35	30	5	68	0.25	20	10	0.40	30	5
24	0.10	20	10	0.35	25	5	69	0.25	20	10	0.40	15	10
25	0.50	20	10	0.30	25	10	70	0.25	20	10	0.40	35	0
26	0.50	20	10	0.30	40	5	71	0.25	20	10	0.10	30	10
27	0.50	20	10	0.30	40	0	72	0.25	20	10	0.10	25	10
28	0.50	20	10	0.10	30	10	73	0.50	50	20	0.30	50	25
29	0.50	20	10	0.10	35	10	74	0.50	50	20	0.30	45	20
30	0.50	20	10	0.10	40	10	75	0.50	50	20	0.20	50	25
31	0.90	20	10	0.80	25	5	76	0.50	50	20	0.20	45	30
32	0.90	20	10	0.80	30	5	77	0.50	50	20	0.10	50	30
33	0.90	20	10	0.70	30	5	78	0.50	50	20	0.10	45	30
34	0.90	20	10	0.70	35	0	79	0.75	50	20	0.85	50	10
35	0.90	20	10	0.60	35	5	80	0.75	50	20	0.60	50	25
36	0.90	20	10	0.60	35	0	81	0.75	50	20	0.60	45	30
37	0.05	40	10	0.20	20	10	82	0.75	50	20	0.60	40	35
38	0.05	40	10	0.20	15	10	83	0.75	50	20	0.85	50	15
39	0.05	40	10	0.30	20	10	84	0.75	50	20	0.85	45	25
40	0.05	40	10	0.30	25	10	85	0.95	50	20	0.85	45	40
41	0.05	40	10	0.40	20	10	86	0.95	50	20	0.85	45	35
42	0.05	40	10	0.40	25	10	87	0.95	50	20	0.60	50	40
43	0.25	20	10	0.40	25	5	88	0.95	50	20	0.60	50	35
44	0.25	20	10	0.40	40	0	89	0.95	50	20	0.50	50	40
45	0.25	20	10	0.50	25	5	90	0.95	50	20	0.50	50	35

Table S3. Lottery pairs (91-180) used in the study.

	Left Lottery			Right Lottery				Left Lottery			Right Lottery		
Pair	*p*	x_1	x_2	*p*	x_1	x_2	*Pair*	*p*	x_1	x_2	*p*	x_1	x_2
91	0.50	20	0	1.00	13	0	136	0.50	30	0	1.00	10	0
92	0.50	20	0	1.00	10	0	137	0.50	30	0	0.10	20	15
93	0.50	20	0	1.00	7	0	138	0.50	30	0	0.10	20	10
94	0.10	40	0	1.00	5	0	139	0.50	30	0	0.25	15	10
95	0.10	40	0	1.00	7	0	140	0.50	30	0	0.25	20	10
96	0.10	40	0	1.00	10	0	141	0.50	30	0	1.00	15	0
97	0.90	40	0	1.00	25	0	142	0.50	30	0	0.90	15	0
98	0.90	40	0	1.00	30	0	143	0.50	30	0	0.90	15	5
99	0.90	40	0	1.00	33	0	144	0.50	30	0	0.75	15	5
100	0.10	20	10	1.00	7	0	145	0.50	30	0	0.75	20	0
101	0.10	20	10	1.00	10	0	146	0.60	30	5	1.00	20	0
102	0.10	20	10	1.00	15	0	147	0.60	30	5	1.00	15	0
103	0.50	20	10	1.00	12	0	148	0.60	30	5	0.25	25	15
104	0.50	20	10	1.00	15	0	149	0.60	30	5	0.25	20	15
105	0.50	20	10	1.00	17	0	150	0.60	30	5	0.10	25	15
106	0.90	20	10	1.00	15	0	151	0.40	30	5	1.00	15	0
107	0.90	20	10	1.00	17	0	152	0.40	30	5	1.00	10	0
108	0.90	20	10	1.00	19	0	153	0.40	30	5	0.75	20	10
109	0.05	40	10	1.00	12	0	154	0.40	30	5	0.75	20	5
110	0.05	40	10	1.00	15	0	155	0.40	30	5	0.90	20	5
111	0.05	40	10	1.00	18	0	156	0.50	50	0	1.00	20	0
112	0.25	20	10	1.00	12	0	157	0.50	50	0	0.10	30	15
113	0.25	20	10	1.00	15	0	158	0.50	50	0	0.10	30	20
114	0.25	20	10	1.00	17	0	159	0.50	50	0	0.25	30	15
115	0.75	20	10	1.00	15	0	160	0.50	50	0	0.25	30	20
116	0.75	20	10	1.00	17	0	161	0.50	50	0	1.00	15	0
117	0.75	20	10	1.00	19	0	162	0.50	50	0	0.90	25	10
118	0.95	40	10	1.00	35	0	163	0.50	50	0	0.90	20	15
119	0.95	40	10	1.00	33	0	164	0.50	50	0	0.75	25	10
120	0.95	40	10	1.00	30	0	165	0.50	50	0	0.75	20	15
121	0.05	50	20	1.00	22	0	166	0.60	50	10	1.00	30	0
122	0.05	50	20	1.00	25	0	167	0.60	50	10	0.10	45	25
123	0.05	50	20	1.00	27	0	168	0.60	50	10	0.10	40	25
124	0.25	20	10	1.00	12	0	169	0.60	50	10	0.25	40	25
125	0.25	20	10	1.00	13	0	170	0.60	50	10	0.25	35	25
126	0.25	20	10	1.00	16	0	171	0.40	50	10	1.00	25	0
127	0.50	50	20	1.00	30	0	172	0.40	50	10	0.90	25	15
128	0.50	50	20	1.00	33	0	173	0.40	50	10	0.90	30	15
129	0.50	50	20	1.00	36	0	174	0.40	50	10	0.75	25	15
130	0.75	50	20	1.00	42	0	175	0.40	50	10	0.75	30	15
131	0.75	50	20	1.00	35	0	176	0.50	40	10	1.00	25	0
132	0.75	50	20	1.00	38	0	177	0.50	40	10	1.00	20	0
133	0.95	50	20	1.00	47	0	178	0.50	40	10	0.25	30	20
134	0.95	50	20	1.00	43	0	179	0.50	40	10	0.75	25	15
135	0.95	50	20	1.00	40	0	180	0.50	40	10	0.10	30	20

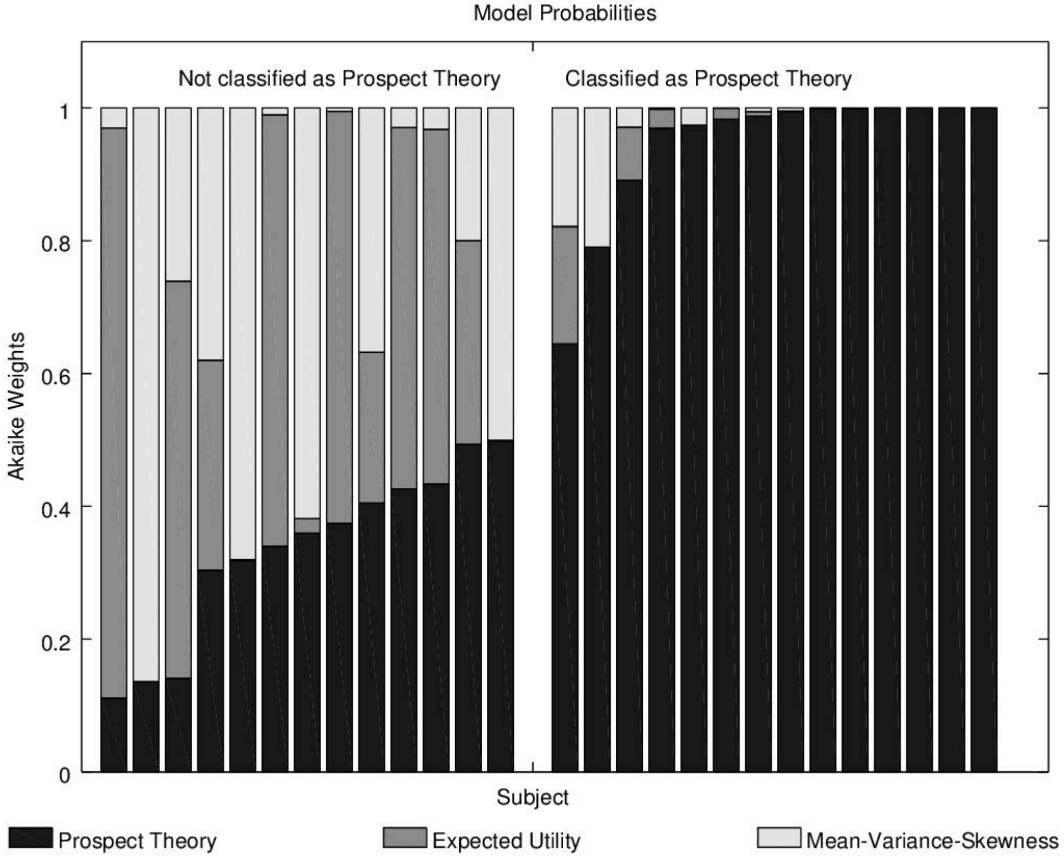

Figure S1. Corrected Akaike weights for the three models in individual participants show that prospect theory is the winning model for only 14 of 27 participants, although it is the winning model over the whole group.

Supplementary analyses

In supplementary analyses, we examined the associations between behavior, the brain regions reported in the main paper, and functional connectivity of these regions with the rest of the brain. These exploratory analyses are not critical for the main findings of the article, but yield additional insights into which brain regions are involved in the choice process triggered by our task and how individual differences in behavioral choice strategies might be associated with connectivity differences in distinct neural networks.

We first used hierarchical linear mixed effects regression to examine the association between the BOLD signal in our regions of interest and response times. This kind of analysis takes random individual variation into account, which makes the effects found on the group-level more robust (14–17). Using extracted BOLD responses from our ROIs and averaging them over each ROI for each participant, we found that the BOLD response in vmPFC is a significant predictor of

response times (β=-.15, t(23.626)=-4.65, p<.001). Thus, the stronger the BOLD response in the vmPFC in a particular trial, the faster the response in that trial, which further strengthens the link of this value difference-representing region to behavior in a general sense. In contrast, the regions representing state value did not show an association with response times.

Next, we assessed how the different models of subjective value related to interactions of our value coding clusters with the rest of the brain, using psychophysiological interaction (PPI) analyses. At the time of choice, the vmPFC region coding absolute value differences showed *differential* functional connectivity depending on which of the three models fitted individual choice behavior best (Expected Utility [EU], Mean-Variance-Skewness [MVS], or Prospect Theory [PT]). This differential connectivity concerned a large cluster comprising left posterior prefrontal, insular, opercular and anterior parietal cortex (peak coordinates: x=-46, y=8, z=-6, F(3,24)=43.37, p_{FWE}<.001, whole-brain voxel-level corrected). For these regions, connectivity with vmPFC was stronger in MVS- compared to both EU- and PT-participants (Figure 1A and B). A similar effect was evident in the right parietal cortex (peak coordinates: x=60, y=-36, z=28, F(3,24)=35.10, p_{FWE}<.001, whole-brain corrected) with strongest positive connectivity in MVS-participants, in line with vmPFC preferentially encoding value difference according to the MVS model (Figure 1A and C). Moreover, the functional connectivity between vmPFC and left anterior parietal cortex was stronger the better the fit of the MVS model to the behavioral data (i.e., the smaller the AICc score; peak coordinates: x=-62, y=-26, z=22, t(25)=-5.88, p_{FWE}<.001 whole-brain cluster-level corrected; Figure 1D). In contrast, connectivity between vmPFC and the right temporal cortex was lower the better the fit of the PT model to the behavioral data (i.e., the smaller the AICc score; peak coordinates: x=58, y=-6, z=-8, t(25)=-5.83, p_{FWE}=.005 whole-brain cluster-level corrected; Figure 1E). These findings demonstrate model-related differences in patterns of functional connectivity and give some indication how PT-like behavior may arise.

Next, we interrogated our functional connectivity findings by assessing the subjective value model-specific parameters as covariates in the analyses. The association of the vmPFC at the moment of choice with temporal and parietal regions increased with the variance weighting (beta sigma) from the MVS model (peak coordinates of left parietal-opercular cluster: x=-60, y=-8, z=6, t(25)=6.16, p_{FWE}=.001; peak coordinates of left temporal cluster: x=-52, y=-12, z=-6, t(25)=5.70, p_{FWE}=.05; peak coordinates of right temporal cluster: x=54, y=-24, z=-2, t(25)=6.40, p_{FWE}<.001; peak coordinates of right parietal-opercular cluster: x=52, y=-2, z=2, t(25)=4.88, p_{FWE}=.09; all results whole-brain cluster-level corrected). Beta sigma reflects the weight given to the variance of outcomes (risk attitude) when computing subjective value according to the MVS model. To assess specificity of these correlations, we considered the rho parameter of PT, which specifies the curvature of the value function, and thereby captures risk attitude in this model. In line with model specificity, all four clusters showed little relation with PT-rho and significantly stronger relations with MVS-beta sigma.

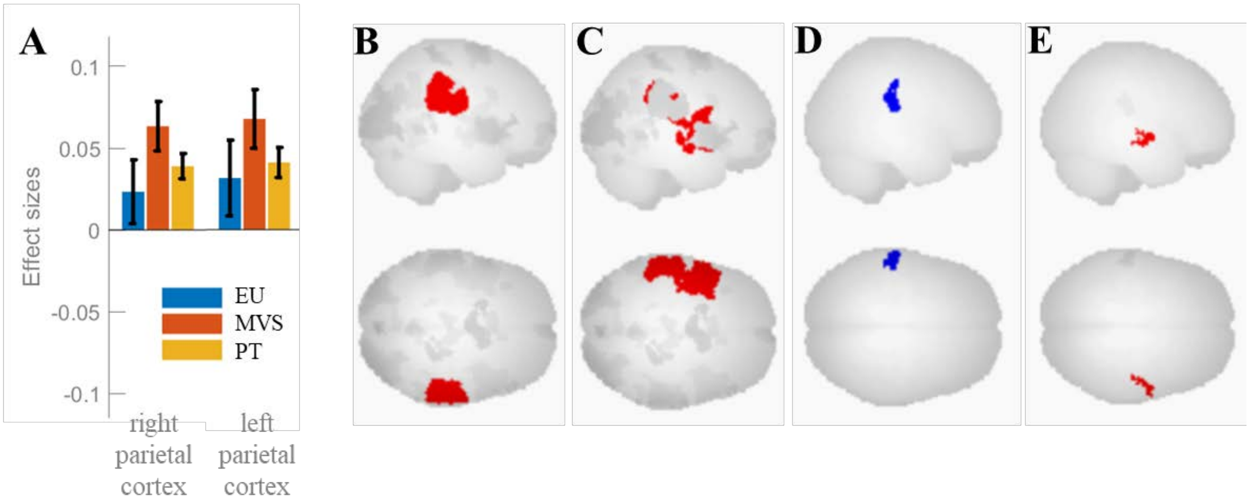

Figure S2. Model-related connectivity of vmPFC. **A** Bar plots showing differential connectivity strengths between vmPFC and frontoparietal clusters depicted in B (left bars) and C (right bars). The bars are separated for participants according to the subjective value model that best fitted the behavioral data (blue = EU, red = MVS, yellow = PT). **B** Cluster in the right parietal and opercular cortex connected more strongly with the vmPFC for MVS than EU- and PT-participants. **C** Cluster in the left parietal, opercular, and insular cortex connected more strongly with the vmPFC for MVS than EU- and PT-participants. **D** Cluster in the left parietal cortex connected more strongly with the vmPFC the better the fit of the MVS model (i.e., the lower the AICc score). **E** Cluster in the right temporal cortex connected less strongly with the vmPFC the better the fit of the PT model (i.e., the lower the AICc score).

To further explore PT-like behavior, we also analyzed functional connectivity in the few trials in which the PT model made a unique behavioral prediction (that is, both EU and MVS predicted choice of one lottery and PT predicted choice of the other lottery) AND participants went with the prediction of the PT model. We used the intraparietal lobule (IPL) and ventral striatum as seeds for these analyses because we found IPL and striatum to be best described as regions processing PT value sums in our model comparisons. PPI analyses revealed differential connectivity of the IPL with a cluster in medial prefrontal cortex (peak coordinates: x=-4, y=56, z=36, $F(2, 25)=33.55$, $p_{FWE}<.001$ whole-brain cluster-level corrected; Figure S2). Specifically, connectivity between these areas was weaker in PT-unique trials when compared to the remaining trials. Interestingly, the mPFC region overlapped with our vmPFC ROI associated with absolute value differences and best explained by the MVS model over the whole sample (Figure S3). Thus, the IPL appears to be connected to the vmPFC in general during value based choices unless the choice follows specifically PT. The finding of reduced connectivity specifically for PT decisions suggests that more local computations, particularly in parietal

cortex, underpin PT-like behavior. This finding converges with reports of a central role of parietal cortex for numeric and arithmetic processing (18) and the stronger requirement for numeric cognition imposed by PT compared to MVS (19).

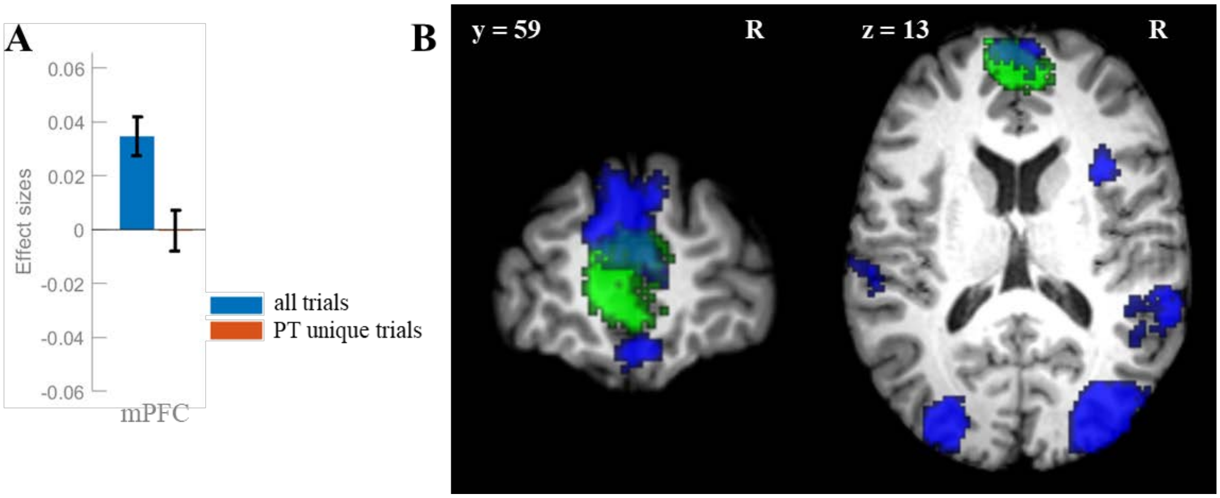

Figure S3. Model-related, behavior-dependent connectivity of IPC. **A** Bar plot showing differential connectivity strengths between the IPC seed and a cluster in mPFC depending on whether PT made different predictions than the other two models. There was little connectivity between these regions at the moment of choice in the trials, in which the PT model uniquely predicted choice (red), while connectivity was positive in the remaining trials (blue). **B** Coronal (left) and axial (right) view of the brain showing the overlap between the cluster positively connected to the IPC seed at time of choice except for the trials that were uniquely predicted by PT (blue) and the vmPFC ROI mask associated most strongly with MVS absolute value differences (green) described in Figure 5.

References

1. K. Blair, *et al.*, Choosing the Lesser of Two Evils, the Better of Two Goods: Specifying the Roles of Ventromedial Prefrontal Cortex and Dorsal Anterior Cingulate in Object Choice. *J. Neurosci.* **26**, 11379–11386 (2006).

2. T. A. Hare, W. Schultz, C. F. Camerer, J. P. O'Doherty, A. Rangel, Transformation of stimulus value signals into motor commands during simple choice. *PNAS* **108**, 18120–18125 (2011).

3. U. Basten, G. Biele, H. R. Heekeren, C. J. Fiebach, How the brain integrates costs and benefits during decision making. *PNAS* **107**, 21767–21772 (2010).

4. S. Palminteri, M. Khamassi, M. Joffily, G. Coricelli, Contextual modulation of value signals in reward and punishment learning. *Nat Commun* **6**, 1–14 (2015).

5. A. Shenhav, M. A. Straccia, M. M. Botvinick, J. D. Cohen, Dorsal anterior cingulate and ventromedial prefrontal cortex have inverse roles in both foraging and economic choice. *Cogn Affect Behav Neurosci* **16**, 1127–1139 (2016).

6. P. S. Hogan, J. K. Galaro, V. S. Chib, Roles of Ventromedial Prefrontal Cortex and Anterior Cingulate in Subjective Valuation of Prospective Effort. *Cereb Cortex* **29**, 4277–4290 (2019).

7. L. Elber-Dorozko, Y. Loewenstein, Striatal action-value neurons reconsidered. *eLife*, 1–32 (2018).

8. T. H. B. FitzGerald, B. Seymour, R. J. Dolan, The Role of Human Orbitofrontal Cortex in Value Comparison for Incommensurable Objects. *J. Neurosci.* **29**, 8388–8395 (2009).

9. A. Shenhav, M. A. Straccia, J. D. Cohen, M. M. Botvinick, Anterior cingulate engagement in a foraging context reflects choice difficulty, not foraging value. *Nat Neurosci* **17**, 1249–1254 (2014).

10. S. B. Eickhoff, *et al.*, A new SPM toolbox for combining probabilistic cytoarchitectonic maps and functional imaging data. *NeuroImage* **25**, 1325–1335 (2005).

11. S. B. Eickhoff, S. Heim, K. Zilles, K. Amunts, Testing anatomically specified hypotheses in functional imaging using cytoarchitectonic maps. *NeuroImage* **32**, 570–582 (2006).

12. S. B. Eickhoff, *et al.*, Assignment of functional activations to probabilistic cytoarchitectonic areas revisited. *NeuroImage* **36**, 511–521 (2007).

13. N. Tzourio-Mazoyer, *et al.*, Automated Anatomical Labeling of Activations in SPM Using a Macroscopic Anatomical Parcellation of the MNI MRI Single-Subject Brain. *NeuroImage* **15**, 273–289 (2002).

14. R. H. Baayen, D. J. Davidson, D. M. Bates, Mixed-effects modeling with crossed random effects for subjects and items. *Journal of Memory and Language* **59**, 390–412 (2008).

15. D. Bates, M. Mächler, B. Bolker, S. Walker, Fitting Linear Mixed-Effects Models Using lme4. *Journal of Statistical Software* **67** (2015).

16. D. P. Janssen, Twice random, once mixed: Applying mixed models to simultaneously analyze random effects of language and participants. *Behav Res* **44**, 232–247 (2012).

17. S. G. Luke, Evaluating significance in linear mixed-effects models in R. *Behav Res* **49**, 1494–1502 (2017).

18. Z. Hawes, H. M. Sokolowski, C. B. Ononye, D. Ansari, Neural underpinnings of numerical and spatial cognition: An fMRI meta-analysis of brain regions associated with symbolic number, arithmetic, and mental rotation. *Neurosci Biobehav Rev* **103**, 316–336 (2019).

19. M. D’Acremont, P. Bossaerts, Neurobiological studies of risk assessment: A comparison of expected utility and mean-variance approaches. *Cognitive, Affective, & Behavioral Neuroscience* **8**, 363–374 (2008).
